# Case 2 / 2018 - Coronary-Cavitary Fistula of Right Ventricular Coronary Artery 5 Years after its Occlusion by Interventional Catheterization

**DOI:** 10.5935/abc.20180048

**Published:** 2018-03

**Authors:** Edmar Atik, Fidel Leal, Raul Arrieta

**Affiliations:** Instituto do Coração (InCor) - Faculdade de Medicina da Universidade de São Paulo, São Paulo, SP - Brazil

**Keywords:** Fistula/congenital, Coronary Vessels, Percutaneous Coronary Intervention

**Clinical data**: Heart murmur detected in routine clinical examination at the age of 8 years, with no other manifestations. The patient was diagnosed as having coronary-cavitary fistula between right coronary artery and right ventricle, which was confirmed by echocardiography. The fistula was then occluded by interventional catheterization, and patient was asymptomatic, with full physical and mental health until the age of 13; in this period, the patient received no drug treatment.

**Physical examination**: good general condition, eupneic, acyanotic, with normal pulse rate at the four limbs. Weight: 36.95 Kg, Height: 154 cm, blood pressure (right arm): 100/60 mm Hg, HR: 76 bpm, oxygen saturation = 97%.

Precordium: apex beat was not palpable, absence of systolic impulses. Normal heart sounds with no heart murmurs. Liver was not palpable.

Before fistula occlusion, apex beat was located at the fifth left intercostal space, with mild systolic impulses at left sternal border and continuous murmurs at mid- and lower left sternal border, intensity grade ++/4, no radiation, and moderately loud heart sounds.

## Complementary tests

**Electrocardiography:** sinus rhythm, with conduction defect in the right bundle branch in the period prior to fistula occlusion. In the late period, there was no evidence of such defect or volume overload.

**Figure1:**
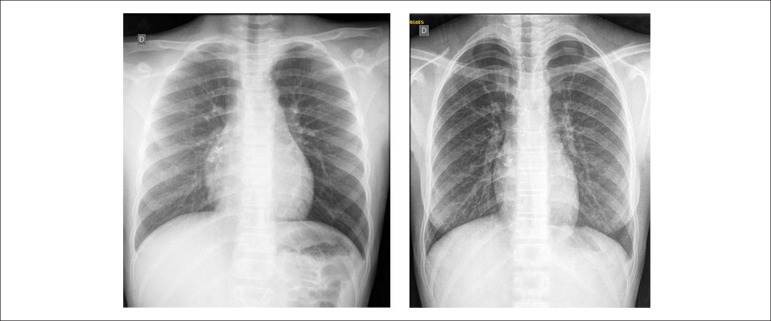
Chest X-rays before (left) and 5 years after (right) coronary-cavitary fistula occlusion, highlighting the decrease in heart size (slightly enlarged before procedure).

**Chest radiography**: slightly increased heart size with cardiothoracic index of 0.47 before the coronary-cavitary fistula occlusion, which was clearly decreased 5 years later (cardiothoracic index 0.41) (Figure 1).

**Echocardiography**: in the period before the coronary-cavitary fistula occlusion, the test revealed dilation of left coronary artery ostium and trunk (8 mm), dilation of circumflex artery (4 mm), and normal anterior descending artery (2 mm). Right coronary artery emerged from the circumflex artery, which was also dilated. Aneurysm in the terminal segment (15 mm) of right coronary artery, before the coronary sinus ostium (4 mm), between the right ventricular outflow and inflow tracts. This chamber was slightly dilated, as well as the right atrium and pulmonary arteries. RV = 20, LV = 35, septum and posterior wall = 6, LA = 23, Ao = 20, RVSP = 20 mmHg, pulmonary arteries = 12 mm. Five years after fistula occlusion, cardiac chambers were normal, and coronary arteries were still dilated despite smaller diameters (left and right coronary trunks of 6 mm and 4 mm, respectively). There was a highly refractive area (10 mm) in the distal third of right coronary artery, corresponding to the arterial plug, and no flow through closed fistula.

**Coronary computed tomography angiography:** Coronary arteries were dilated, with left coronary artery main trunk of 7 mm-diameter, circumflex artery and right coronary artery of 6 mm with its distal end in the right ventricle.

**Clinical diagnosis:** coronary-cavitary fistula of right ventricular coronary artery, of little clinical repercussion, but with important dilation of the coronary circulation. Coronary dilation persisted even after fistula occlusion.

**Clinical reasoning:** There was clinical evidence of coronary-cavitary fistula, related to the presence of continuous murmurs at mid- and lower left sternal border. Due to this condition, the systemic fistula was supposed to occur in the right atrium or ventricle and was of minor clinical relevance, due to the modest increase in right cardiac chambers, revealed by echocardiography. The diagnosis was also established by coronary computed tomography angiography.

**Differential diagnosis:** In asymptomatic patients with continuous murmurs at lower left sternal border, differential diagnosis should include other types of communications between the systemic and pulmonary circulation, such as the aortopulmonary window between the ascending aorta and pulmonary trunk, and fistulas between the Valsalva aortic sinus and right cardiac chambers. When these communications are in the left ventricle, and anastomosis with the left atrium occurs, the murmur is diastolic and continuous, and heard in other regions, cardiac apex and axilla.

**Figure 2:**
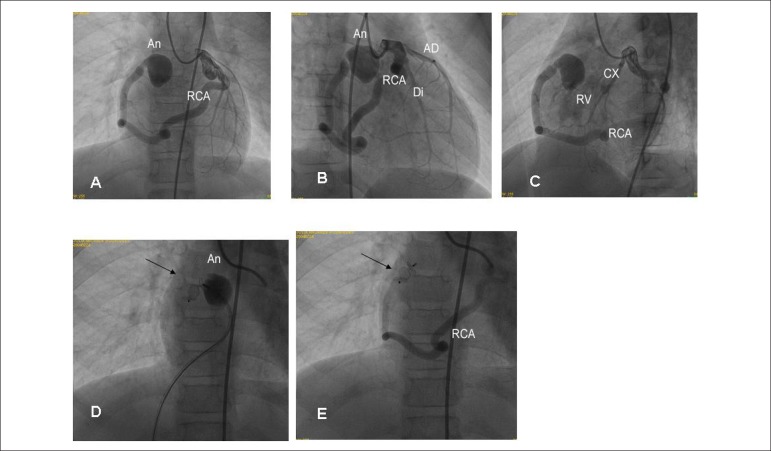
Coronary cineangiography showing important dilation of right coronary artery (RCA), emerging from the circumflex artery and terminating in the aneurysmal compartment (A and B). Drainage of aneurysm in the final segment of RCA was conducted in the right ventricle (RV). Insertion of the Amplatzer vascular plug II (arrow) from the RV can be seen in RCA, in the segment anterior to the coronary aneurysm (D) and interruption of the fistula drainage (E). Cx: circumflex; Di: diagonalis; AD: anterior descending artery.

**Management:** The treatment of choice for coronary-cavitary fistula accompanied by dilation of coronary arteries was interventional catheterization. Coronary artery had a 6 mm-diameter, with aneurysm in its terminal segment of approximately 15 mm, and a 4-mm communication with the right ventricle. Occlusion of the aneurysm was successfully performed using a Amplatzer vascular plug II, with immediate resolution of the fistula. (Figure 2).

**Comments:** Rare congenital coronary artery fistulas are abnormal communications with cardiac cavities or the pulmonary arterial tree. Drainage is more commonly performed in the right cavities and occasionally in coronary sinus or left cavities. These fistulas may be simple or multiple, and cause a proportional volume overload, mimicking conditions including interatrial communication, interventricular communication and arterial channel persistence, depending on the drainage site. In addition, they cause myocardial ischemia, arrhythmias, vascular rupture, and endocarditis. Therefore, and effective treatment of these fistulas is paramount, and has been performed by surgery or by interventional catheterization since 1983.^1^ Positive results of both procedures overcome complications which include infarction, prosthesis embolization, fistula dissection and arrhythmia. Indication for percutaneous intervention increases in face of a faster recovery, lower morbidity and lower cost. It is of note that coronary artery dilation is not reduced even after fistula resolution, which reflects the presence of concomitant lesion of elastic fibers of the vessel, that surpasses its limits of distensibility.
